# Prediction of Cyanotic Congenital Heart Disease Risk in U.S. Births

**DOI:** 10.3390/jcdd13050178

**Published:** 2026-04-25

**Authors:** Riya Reddy, Marwan Saad, Frank W. Sellke

**Affiliations:** 1 Cardiovascular Research Center, Division of Cardiothoracic Surgery, Rhode Island Hospital, Providence, RI 02903, USA; 2 Department of Medicine, Division of Cardiology, Brown University Health, Providence, RI 02903, USA; 3 Department of Surgery, Division of Cardiothoracic Surgery, Warren Alpert Medical School, Brown University, Providence, RI 02903, USA

**Keywords:** cyanotic congenital heart disease (CCHD), maternal factors, social determinants of health

## Abstract

Cyanotic congenital heart disease (CCHD) remains a major contributor to infant morbidity and mortality in the United States, yet the influence of maternal social determinants of health on risk is not fully understood. This study examined associations of maternal age, education, and race/ethnicity with the live birth prevalence of CCHD using recent national birth data from the Centers for Disease Control and Prevention (CDC) National Vital Statistics System (2022–2023). CCHD was identified from birth certificate records and analyzed as a binary outcome. Regression analyses were performed to evaluate relationships between maternal characteristics and CCHD occurrence. Overall, CCHD was a rare outcome with a modest decline in prevalence between the two years examined. Increasing maternal age was associated with higher odds of CCHD, while Latina ethnicity was associated with lower odds compared to the reference group. Other racial/ethnic categories and maternal education level were not significantly associated with CCHD risk in adjusted analyses. These findings suggest that certain maternal factors, particularly age and ethnicity, are associated with variation in the live birth prevalence of CCHD and underscore the need for further research into underlying environmental and structural contributors not captured in standard birth records.

## 1. Introduction

Congenital heart disease (CHD), the most prevalent category of major congenital malformations worldwide, represents a major public health challenge and remains the leading cause of mortality among infants and young children from non-communicable diseases [1]. CHD affects approximately 1% of live births globally, which is roughly 1.35 million new cases annually [2]. In the United States alone, approximately 40,000 infants are born with CHD each year, and the total population living with these conditions has reached 2.4 million, including 1.4 million adults [3]. Cyanotic congenital heart disease (CCHD) encompasses defects characterized by right-to-left shunting and resultant systemic hypoxemia. These lesions are typically life-threatening and often require surgical or catheter-based intervention within the neonatal period or early infancy [4]. In contrast, acyanotic congenital heart defects comprise a heterogeneous group of lesions that typically maintain systemic oxygenation. These include left-to-right shunt lesions associated with increased pulmonary blood flow, as well as obstructive lesions that do not involve pulmonary overcirculation. This study focuses specifically on CCHD as it represents a more severe subset of defects. Given its clinical urgency and higher morbidity and mortality, understanding population-level risk factors for CCHD may have greater implications for prenatal risk stratification and early detection compared to acyanotic lesions.

Despite improved outcomes driven by advances in diagnosis and surgical care, the existing literature suggests that the etiology of most congenital heart disease remains unclear and likely reflects a complex interplay of genetic and environmental influences [5]. The relationship between maternal factors and cyanotic congenital heart disease (CCHD) is particularly strong, with specific environmental exposures and health behaviors showing strong associations with this severe phenotype [6]. Established maternal clinical conditions, including pre-gestational diabetes, hypertension, and phenylketonuria, are recognized risk factors for CHD [6]. Beyond these clinical risk factors, increasing attention has been directed toward the role of social and structural determinants in shaping disease risk. Emerging evidence suggests that maternal race and participation in socioeconomic support programs such as the Special Supplemental Nutrition Program for Women, Infants, and Children (WIC) have a statistically significant impact on CCHD incidence [7]. Fundamental social causes shape disease risk by influencing environmental exposures, health behaviors, and access to resources, ultimately altering susceptibility to congenital abnormalities [8]. In the field of congenital cardiology, social determinants of health are known to influence short-term and long-term outcomes [9]. However, the role of social determinants in the development of congenital heart disorders is not well understood. In particular, less is known about how maternal social determinants specifically relate to cyanotic congenital heart disease, a more severe and clinically distinct subgroup, and recent national-level analyses using contemporary U.S. data remain limited. Therefore, this study aims to evaluate the association of maternal age, education, and race/ethnicity with the live birth prevalence of CCHD using recent national U.S. data.

In previous studies, maternal education has emerged as an important predictor of CHD risk and outcomes, with lower educational attainment associated with higher incidence of both CHD and CCHD [10]. It has been consistently shown that advanced maternal age (≥35 years) increases CHD risk primarily through chromosomal aneuploidies [11]. Some studies suggest that advanced maternal age, as an independent risk factor, is associated with a modest increase in CHD among mothers older than 40 years [12,13]. Prior population level analyses have identified racial and ethnic disparities in CCHD, although findings are heterogeneous and depend on underlying population characteristics and adjustment for clinical and socioeconomic factors [6]. The “Latino health paradox” is a well-documented phenomenon in which Latina women demonstrate relatively favorable perinatal outcomes despite socioeconomic disadvantage [14]. Studies have demonstrated that Hispanic women often have comparable or improved perinatal outcomes, including lower rates of adverse birth outcomes, relative to non-Hispanic White populations, although these patterns vary by subgroup and maternal nativity [15].

Prenatal identification of high-risk fetuses enables coordinated delivery planning and resource allocation, and supports optimization of maternal health [16]. Maternal cardiovascular risk factors extend beyond the perinatal period, contributing to adverse cardiovascular profiles in offspring that extend into adolescence and perpetuate a cycle of cardiovascular risk [17,18]. Consequently, understanding social and maternal determinants associated with the likelihood of CCHD may help inform preventive strategies, support early screening and timely diagnosis, and guide future efforts toward targeted prenatal or in utero interventions aimed at improving both maternal and infant outcomes.

## 2. Materials and Methods

The database used to explore this research question was the CDC’s National Vital Statistics System (NVSS). The birth certificates in this dataset clearly list congenital abnormalities, including cyanotic congenital heart disease. A 10% random sample of birth records from each year (2022–2023) was used to facilitate efficient data handling while preserving a large, nationally representative sample. Random sampling minimizes selection bias and ensures that the sample reflects the underlying population distribution, thereby supporting valid and generalizable statistical inference. Considering the low prevalence of CCHD, the resulting sample size remained sufficiently large to ensure adequate statistical power and stable regression estimates. The NVSS dataset was unable to stratify the types of cyanotic congenital heart defects present, limiting the ability to conduct a more detailed analysis. However, given that very specific types of cyanotic congenital heart defects are incredibly rare, conducting such an analysis would likely not yield interpretable results. The United States was the geographic region of study, and this provided a greater sample to perform analysis on compared to using only specific regions in the country, since cyanotic congenital heart disease is an uncommon variable. The time period examined was 2022–2023 as it had the most recent data available. Incorporating this time with a national perspective allowed for capturing current trends and applicable correlations, and making accurate predictions for the future.

The sample was designed to account for three primary variables: maternal age, maternal education, and maternal race. Birth certificate data were restricted to mothers aged 18–50 years, as prior studies have shown increased CHD risk with maternal age over 40 [12,13]. Maternal race was categorized as non-Hispanic White, non-Hispanic Black, Latina, American Indian, and Asian, with White serving as the reference group. Race has been associated with CHD incidence and outcomes, with racial minorities often experiencing higher rates and poorer prognoses due to structural inequities. Maternal education was categorized into less than high school, high school, some college, and college degree to capture meaningful distinctions in socioeconomic position. Education was considered a proxy for broader social determinants of health, as lower educational attainment often coincides with limited access to prenatal care and other risk-enhancing conditions. The outcome variable was the presence or absence of cyanotic congenital heart disease, coded as binary, with 1 = CCHD present, and 0 = CCHD not present. All analyses were conducted using Stata/SE 18.0, with statistical significance set at *p* < 0.05.

Descriptive summary statistic tables were used to summarize the data, and linear and logistic regression tables were utilized for further analysis. Regression analyses were performed for predicting cyanotic congenital heart disease based on the predictors maternal age, maternal education, and maternal race. Linear regression was initially used to hold the other factors constant and examine general associations between maternal characteristics and CCHD. However, CCHD is a rare binary outcome, so logistic regression was also conducted to account for the non-linear nature of such events. This approach allowed for assessing whether the results remained consistent when the rarity of the outcome was properly modeled. Graphs illustrating the correlation between all variables were also generated. These included a bar graph showing CCHD probability by maternal race, a bar graph displaying CCHD probability by education level, and a line graph depicting the probability of CCHD across maternal age.

## 3. Results

The data included births that were mostly evenly split between 2022 and 2023 (mean = 2022.495, std. dev. = 0.499). Cyanotic congenital heart disease was a very rare outcome with limited variation, as only 0.06% of the observed births were affected by CCHD across a sample of 718,920 observations (mean = 0.0006, std. dev. = 0.025). Maternal age is presented separately as a continuous variable and is not included in Table 1. The majority of mothers were in their late 20s to early 30s (mean = 29.68 years, std. dev. = 5.68). Most mothers had at least a high school education, with the largest share having a bachelor’s degree (10.5% had less than a high school education, 26.8% had completed high school, 26.4% had some college education, and 36.4% held a bachelor’s degree). White mothers made up much of the sample, followed by Latina and Black mothers, while American Indian and Asian mothers represent smaller portions of the population (52.0% White, 14.1% Black, 0.7% American Indian, 6.7% Asian, and 26.5% Latina).

For each one-year increase in maternal age (between 18 and 50), there is a very small but statistically significant increase in the probability of the child having CCHD (*p* = 0.007). Although the effect size is small, the association is statistically meaningful (Table 2). None of the maternal education levels show a statistically significant association with cyanotic congenital heart defects when compared to the reference category. This suggests that maternal education level may not be a strong predictor in this model. Children of Latina mothers have a statistically significant lower probability of CCHD compared to children of White mothers. Other race categories do not show statistically significant differences. Infants born in 2023 are slightly less likely to have cyanotic congenital heart defects compared to those born in 2022, controlling for maternal age, education, and race (*p* = 0.010). Although the effect is very small, it is statistically significant.

Many maternal factors are interrelated (e.g., race and education). Logistic regression controls for these overlapping effects, isolating the independent association between each variable and CCHD risk (Table 3). Logistic regression does not assume normality; instead, it assumes a log-linear relationship between predictors and the log odds of the outcome, making it an ideal test for binary variables. Each additional year of maternal age is associated with a 2.5% increase in the odds of cyanotic congenital heart defects (confidence interval: 1.0068–1.0434). Being Latina is associated with approximately 36% lower odds of congenital heart defects compared to White mothers, who served as the reference group. Additionally, the odds of congenital heart defects in 2023 were 22% lower than in 2022.

**Figure 1:**
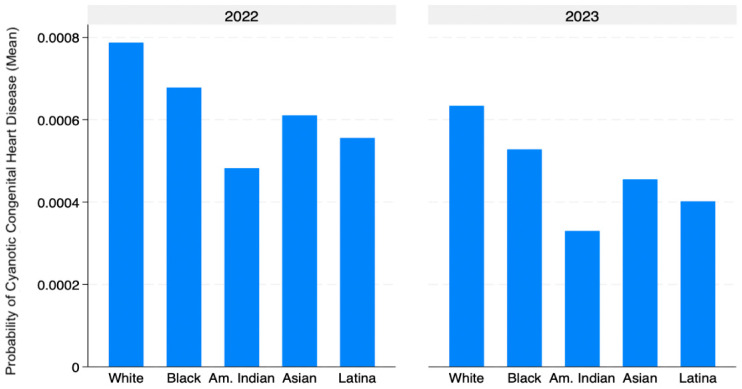
Probability of cyanotic congenital heart disease by maternal race.

White mothers had the highest probability of CCHD outcomes in their children, with an estimated probability of approximately 0.0008. Black and Asian mothers followed, each with moderately high probabilities. American Indian mothers had the lowest probability of CCHD, while Latina mothers had slightly lower probabilities than Asian mothers but higher than American Indian mothers. Across all groups, probabilities slightly declined in 2023 (Figure 1).

**Figure 2:**
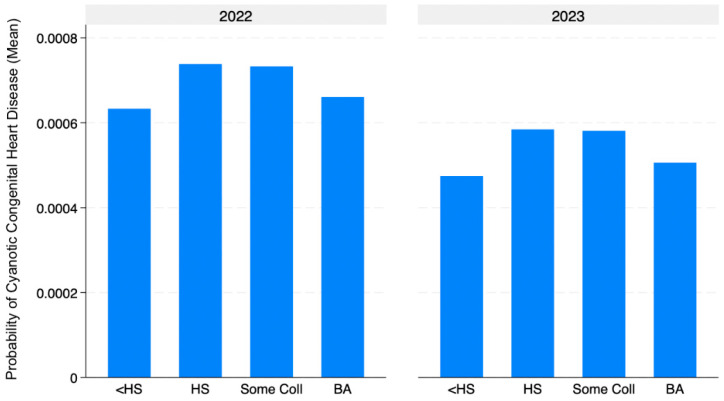
Probability of cyanotic congenital heart disease by maternal education.

The highest probabilities of CCHD were observed among mothers with high school diplomas and some college education. In contrast, the lowest probabilities were seen among mothers with less than a high school education and those with bachelor’s degrees. This pattern suggests a non-linear relationship between maternal education and CCHD risk, with moderate education levels associated with slightly higher risk compared to the extremes. A slight overall decrease in CCHD probability was noted across all education levels (Figure 2).

**Figure 3:**
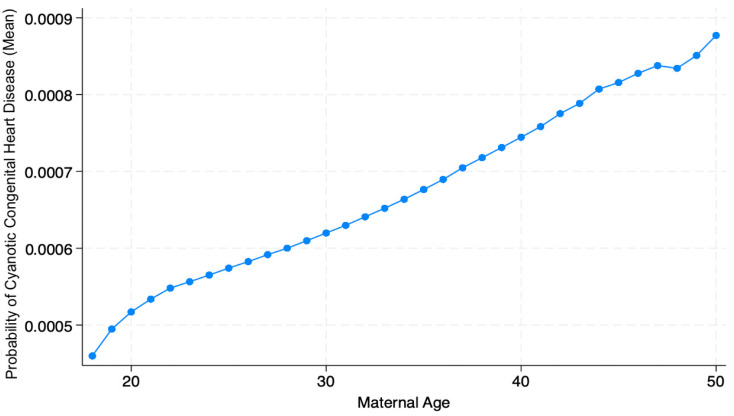
Probability of cyanotic congenital heart disease by maternal age.

Figure 3 suggests a steady increase in the probability of CCHD as maternal age rises. Between ages 18 and approximately 50, the probability of CCHD climbs from about 0.00045 to nearly 0.0009, effectively doubling over time. An inflection point appears around ages 35 to 40, where the slope becomes slightly steeper, consistent with established medical evidence that advanced maternal age (35 and older) is a recognized risk factor for fetal anomalies, including CCHD. Overall, the chart supports the clinical understanding that the risk of congenital anomalies, including CCHD, increases progressively with maternal age.

Descriptive analyses demonstrated variation in the unadjusted probability of CCHD across maternal age, education, and race. White mothers exhibited the highest observed probability of CCHD, followed by Black and Asian mothers, while Latina and American Indian mothers showed lower probabilities. Similarly, CCHD probability increased with maternal age and varied modestly across education levels. However, these descriptive probabilities reflect unadjusted associations. In adjusted regression analyses, maternal age remained significantly associated with increased odds of CCHD, with each additional year corresponding to a 2.5% increase in odds. Latina ethnicity was associated with lower odds of CCHD compared to White ethnicity, while other racial/ethnic groups and maternal education were not consistently associated with CCHD risk. Although differences were observed in unadjusted probabilities, not all patterns persisted after adjustment, highlighting the importance of regression analyses in identifying independent associations by accounting for confounding variables.

## 4. Discussion

These findings suggest that demographic factors such as maternal age and race/ethnicity are associated with variation in the live birth prevalence of CCHD, while maternal education showed a weaker relationship. These associations likely reflect complex interactions between biological, structural, and healthcare-related factors rather than direct causal effects of individual characteristics.

Consistent with previous research, descriptive analyses demonstrated a positive association between maternal age and the unadjusted probability of CCHD, with increasing maternal age corresponding to higher observed probabilities. In adjusted regression analyses, maternal age remained significantly associated with increased odds of CCHD, supporting existing literature that identifies advanced maternal age as a risk factor for congenital anomalies, potentially due to chromosomal instability and increased incidence of maternal comorbidities such as hypertension and diabetes [6,13]. The lack of increased risk among the youngest maternal age group suggests that age-related CCHD risk is primarily concentrated among older mothers. Although advanced maternal age is a well-established risk factor for congenital heart disease, our findings demonstrate that this association persists in contemporary national data and is specifically observed in CCHD. This reinforces the continued relevance of maternal age in modern risk stratification and prenatal screening practices.

Although mothers with high school diplomas and some college education had slightly higher unadjusted probabilities of CCHD compared to those with less than high school or bachelor’s degrees, these differences were very little. In adjusted regression models, maternal education did not demonstrate a consistent independent association with the odds of CCHD. This suggests that maternal education, when considered in isolation, may not be a strong independent predictor of CCHD risk. As such, reliance on education alone as a proxy for socioeconomic status may obscure more meaningful underlying associations. Focusing on more clear economic factors (i.e., income level and housing security) may yield different results.

Descriptive analyses of unadjusted probabilities showed that White mothers had the highest observed probability of CCHD, while Latina and American Indian mothers had lower observed probabilities across both 2022 and 2023. However, these patterns reflect unadjusted associations and should be interpreted with caution. In adjusted regression analyses, Latina ethnicity remained associated with lower odds of CCHD compared to White ethnicity, while other racial and ethnic groups did not demonstrate consistent independent associations. These findings differ from some prior studies reporting higher CCHD burden among minority populations, but they are consistent with the heterogeneity observed in the literature [6,19].

The lower observed live birth prevalence of CCHD among Latina mothers may reflect the “Latino health paradox,” a well-documented epidemiologic phenomenon in which Latina women demonstrate relatively favorable perinatal outcomes despite socioeconomic disadvantage [14]. This paradox has been consistently observed across population-based studies, with Hispanic mothers exhibiting lower rates of adverse birth outcomes such as low birth weight and infant mortality compared to non-Hispanic White populations, particularly among foreign-born individuals [15,20]. Although this paradox has been well described in relation to general perinatal outcomes, its relevance to CHD remains less well understood. The present findings suggest that at the population level, similar patterns may extend to the live birth prevalence of CCHD. The observed differences may also reflect unmeasured clinical and structural factors, including variation in maternal comorbidities, access to prenatal care, and differences in prenatal diagnosis and pregnancy continuation. These differences were not captured in the NVSS dataset. Furthermore, the Latino population is heterogeneous, and these patterns may differ by country of origin and socioeconomic context. Taken together, these findings highlight the need for further investigation into how social, behavioral, and structural determinants contribute to variation in CCHD risk and outcomes.

While this pattern has been well described in studies of birth outcomes, its relevance to congenital heart disease remains less well understood. The present findings therefore provide important population level evidence suggesting that similar patterns may extend to the live birth prevalence of CCHD, highlighting a potential area for further investigation.

The observed decrease in CCHD prevalence from 2022 to 2023 was statistically significant, with a notable relative reduction in odds but a small absolute change in prevalence given the rarity of the outcome. Still, it is an unexpected finding that warrants further investigation. However, the current dataset does not include variables that would allow for identification of the underlying causes of this change. As such, this finding should be interpreted with caution. Potential contributing factors, such as changes in prenatal screening practices, healthcare access, or reporting patterns, were not evaluated in this study. Further research incorporating longitudinal data and additional clinical and structural variables is needed to better understand temporal trends in CCHD prevalence.

### Limitations

The dataset did not stratify based on different types of congenital heart disease, limiting the ability to explore associations with specific subtypes. Only cyanotic congenital heart disease was explicitly specified on birth certificates, while other types were grouped together. The range of variables available on birth certificates was limited, restricting the ability to control for potential confounders or conduct more nuanced analyses. It is important to note that congenital heart disease is a very rare outcome, which may limit statistical precision and interpretation. The NVSS dataset includes limited maternal clinical information and does not comprehensively capture key risk factors, including conditions such as phenylketonuria, which may result in residual confounding.

An important limitation of this study is that the analysis is restricted to live birth data and does not capture pregnancies affected by CCHD that result in miscarriage or termination. As a result, the findings reflect live birth prevalence, which may differ from total fetal incidence. Differences in observed prevalence across racial and ethnic groups may therefore be influenced by variation in prenatal detection, access to diagnostic services, and pregnancy-related decision making. Furthermore, the analysis was limited to two years of data (2022–2023), and while a difference between years was observed, this should not be interpreted as a stable temporal trend but rather as a preliminary observation that warrants further investigation using longitudinal data.

Future research would benefit from more detailed clinical data to better characterize specific types of congenital heart defects. To gain a more comprehensive understanding of CHD outcomes, additional variables related to healthcare access, socioeconomic status, maternal behaviors, and structural factors should be incorporated into the analysis.

## 5. Conclusions

This study investigated how maternal age, education, and race relate to the live birth prevalence of CCHD in newborns. Maternal age, Latina ethnicity, and year of birth were statistically significantly associated with the odds of cyanotic congenital heart defects. Most education and other race variables were not consistently associated with CCHD in adjusted analyses. Given the rarity of CCHD, the overall predictive power of the model was limited, suggesting that additional clinical, environmental, and structural factors not captured in this dataset may contribute to disease risk.

Targeted screening based on maternal age may be particularly valuable, particularly given the increased risk of CCHD associated with advancing maternal age. Enhanced screening protocols for pregnancies involving older mothers, especially those over age 35, are already in place, and this study supports the continued relevance of such approaches. Implementing maternal age-based risk stratification policies that allocate more intensive monitoring and resources to older mothers could improve early detection and intervention for CCHD. Further refinement of maternal age-based risk stratification may help inform early detection and intervention efforts for CCHD. It is important to avoid making assumptions based solely on maternal education, as education levels do not show a direct relationship with CCHD risk.

This study provides population-level insight into how maternal demographic factors are associated with variation in the live birth prevalence of CCHD, but these findings should be interpreted within the context of the study’s limitations, including the use of live birth.

Future research that explores more detailed clinical subtypes of cyanotic congenital heart disease, as well as a broader range of social and structural determinants, may provide a more comprehensive understanding of CCHD risk. In particular, understanding other variables (e.g., housing, access to care, insurance coverage, etc.) would provide greater insights. Incorporating longitudinal and clinically detailed data will be important for clarifying underlying mechanisms and improving risk prediction. Research that expands upon this study would enable more comprehensive interpretation of CCHD outcomes.

## Figures and Tables

**Table 1:** Descriptive statistics of study population.

Variable	Number of Pregnancies Assessed	Category	Proportion
CCHD	718,920	Yes	0.06%
		No	99.4%
Maternal Education	708,033	Less than high school	10.5%
		High school	26.8%
		Some college	26.4%
		Bachelor’s degree	36.4%
Maternal Race	702,768	White	52.0%
		Black	14.1%
		American Indian	0.7%
		Asian	6.7%
		Latina	26.5%
Calendar Year	720,501	2022	49.9%
		2023	50.1%

Baseline characteristics of the study population. Values are presented as proportions (%) of total observations within each category for maternal education, race/ethnicity, CCHD status, and calendar year.

**Table 2:** Linear regression of cyanotic congenital heart disease (CCHD).

Variable	Coefficient	Std. Err.	*t*	*p* > |*t*|	[95% Conf. Interval]
Maternal Age	0.0000155	5.77 × 10^−6^	2.69	0.007 **	[4.20 × 10^−6^, 0.0000268]
Maternal Education <HS Omitted					
HS	0.0000783	0.0001103	0.71	0.478	[−0.0001378, 0.0002945]
Some College	0.0000148	0.0001117	0.13	0.894	[−0.0002042, 0.0002338]
BA	−0.0001404	0.0001129	−1.24	0.214	[−0.0003618, 0.0000809]
Maternal Race (White Omitted)					
Black	−0.0001423	0.0000917	−1.55	0.121	[−0.0003221, 0.0000375]
American Indian	−0.0003371	0.0003585	−0.94	0.347	[−0.0010398, 0.0003656]
Asian	−0.0001791	0.0001257	−1.42	0.154	[−0.0004254, 0.0000673]
Latina	−0.0002615	0.0000752	−3.48	0.001 **	[−0.000409, −0.000114]
Year (2022 as a Reference)	−0.0001539	0.00006	−2.56	0.010 *	[−0.0002715, −0.0000362]
Constant	0.311453	0.1214261	2.56	0.010 *	[0.0734619, 0.5494442]

Abbreviations: Std. Err. = standard error; *t* = *t*-statistic; *p* > |*t*| = *p*-value for the *t*-test of statistical significance; 95% Conf. Interval = 95% confidence interval. Coefficients represent estimated changes in the outcome variable per unit increase in the predictor. Reference categories: <HS for maternal education, White for maternal race, and 2022 for year. * *p* < 0.05, ** *p* < 0.01.

**Table 3:** Logistic regression of cyanotic congenital heart disease (CCHD).

Variable	Odds Ratio	Std. Err.	z	*p* > |z|	[95% Conf. Interval]
Maternal Age	1.025	0.009	2.70	0.007 **	[1.006752, 1.043376]
Maternal Education <HS Omitted					
HS	1.134	0.209	0.69	0.493	[0.7912346, 1.6265831
Some College	1.026	0.191	0.14	0.892	[0.7121246, 1.476816]
BA	0.804	0.152	−1.15	0.249	[0.555128, 1.164596]
Maternal Race (White Omitted)					
Black	0.803	0.118	−1.49	0.137	[0.6022321, 1.071912]
American Indian	0.543	0.386	−0.86	0.391	[0.1349091, 2.189545]
Asian	0.748	0.161	−1.35	0.177	[0.4904824, 1.140035]
Latina	0.643	0.083	−3.42	0.001 **	[0.4988034, 0.8280904]
Year (2022 as a Reference)	0.779	0.076	−2.56	0.011 *	[0.643327, 9432405]
Constant	8.7 × 10^215^	1.7 × 10^218^	2.52	0.012 *	[7.68 × 10^47^, infinity]

Abbreviations: Std. Err. = standard error; z = z-statistic; *p* > |z| = *p*-value for the z-test of statistical significance; 95% Conf. Interval (CI) = 95% confidence interval. Odds ratios represent the change in odds of CCHD per unit increase in the predictor. Reference categories: <HS for maternal education, White for maternal race, and 2022 for year. * *p* < 0.05, ** *p* < 0.01.

## Data Availability

The original data presented in the study are openly available in the CDC National Vital Statistics System (Years 2022–2023) at this link: https://www.cdc.gov/nchs/data_access/vitalstatsonline.htm (accessed on 10 March 2025).

## Abbreviations

The following abbreviations are used in this manuscript:

CCHD	Cyanotic Congenital Heart Disease
CHD	Congenital Heart Disease
NVSS	National Vital Statistics System
CDC	Centers for Disease Control

## References

[1] Xu J., Li Q., Deng L., Xiong J., Cheng Z., Ye C. (2025). Global, regional, and national epidemiology of congenital heart disease in children from 1990 to 2021. Front. Cardiovasc. Med..

[2] Van Der Linde D., Konings E.E.M., Slager M.A., Witsenburg M., Helbing W.A., Takkenberg J.J.M., Roos-Hesselink J.W. (2011). Birth prevalence of congenital heart disease worldwide. J. Am. Coll. Cardiol..

[3] (2025). Data and Statistics. Congenital Heart Defects (CHDs). https://www.cdc.gov/heart-defects/data/index.html.

[4] Cleveland Clinic (2025). Cyanotic Heart Disease. https://my.clevelandclinic.org/health/diseases/22441-cyanotic-heart-disease.

[5] Deng L., Li Q., Cheng Z. (2025). Evaluating the global, regional, and national burden of congenital heart disease in infants younger than 1 year: A 1990–2021 systematic analysis for the GBD study 2021. Front. Pediatr..

[6] Ebeh D.N., Jahanfar S. (2021). Association between maternal race and the occurrence of cyanotic congenital heart disease in the USA. SN Compr. Clin. Med..

[7] Shahid S., Khurram H., Lim A., Shabbir M.F., Billah B. (2024). Prediction of cyanotic and acyanotic congenital heart disease using machine learning models. World J. Clin. Pediatr..

[8] Link B.G., Phelan J. (1995). Social Conditions as Fundamental Causes of Disease. J. Health Soc. Behav..

[9] Peyvandi S., Baer R.J., Chambers C.D., Norton M.E., Rajagopal S., Ryckman K.K., Moon-Grady A., Jelliffe-Pawlowski L.L., Steurer M.A. (2020). Environmental and Socioeconomic Factors Influence the Live-Born Incidence of Congenital Heart Disease: A Population-Based Study in California. J. Am. Heart Assoc..

[10] Tran M., Miner A., Merkel C., Sakurai K., Woon J., Ayala J., Nguyen J., Lopez J., Friedlich P., Votava-Smith J.K. (2023). Sociodemographic profile associated with congenital heart disease among infants <1 year old. Res. Sq..

[11] Jenkins K.J., Botto L.D., Correa A., Foster E., Kupiec J.K., Marino B.S., Oster M.E., Stout K.K., Honein M.A. (2019). Public health approach to improve outcomes for congenital heart disease across the life span. J. Am. Heart Assoc..

[12] Liu S., Joseph K.S., Lisonkova S., Rouleau J., Hof M.V.D., Sauve R., Kramer M.S. (2013). Association between maternal chronic conditions and congenital heart defects. Circulation.

[13] Nørregaard M.M.O., Basit S., Sillesen A.S., Raja A.A., Jørgensen F.S., Iversen K.K., Bundgaard H., Boyd H.A., Vøgg R.O.B. (2023). Impact of maternal age and body mass index on the structure and function of the heart in newborns: A Copenhagen Baby Heart Study. BMC Med..

[14] McGlade M.S., Saha S., Dahlstrom M.E. (2004). The Latina paradox: An opportunity for restructuring prenatal care delivery. Am. J. Public Health.

[15] Montoya-Williams D., Williamson V.G., Cardel M., Fuentes-Afflick E., Maldonado-Molina M., Thompson L. (2020). The Hispanic/Latinx Perinatal Paradox in the United States: A scoping review and recommendations to guide future research. J. Immigr. Minor. Health.

[16] Patel S.R., Michelfelder E. (2024). Prenatal diagnosis of congenital heart disease: The crucial role of perinatal and delivery planning. J. Cardiovasc. Dev. Dis..

[17] Hossin M.Z., Kazamia K., Faxén J., Rudolph A., Johansson K., Sandström A., Razaz N. (2024). Pre-existing maternal cardiovascular disease and the risk of offspring cardiovascular disease from infancy to early adulthood. Eur. Heart J..

[18] Zhang D.W., Zhu Y.B., Zhou S.J., Chen X.-H., Li H.-B., Liu W.-J., Wu Z.-Q., Chen Q., Cao H. (2024). Maternal cardiovascular health in early pregnancy and the risk of congenital heart defects in offspring. BMC Pregnancy Childbirth.

[19] Rauscher E., Rangel D.E. (2020). Rising Inequality of Infant Health in the U.S. SSM-Popul. Health.

[20] Giuntella O. (2016). The Hispanic health paradox: New evidence from longitudinal data on second and third-generation birth outcomes. SSM-Popul. Health.

